# Recruitment of Thermogenic Fat: Trigger of Fat Burning

**DOI:** 10.3389/fendo.2021.696505

**Published:** 2021-07-22

**Authors:** Zhihan Wang, Xuefeng Yu, Yong Chen

**Affiliations:** ^1^ Division of Endocrinology, Internal Medicine, Tongji Hospital, Huazhong University of Science & Technology, Wuhan, China; ^2^ Laboratory of Endocrinology, Tongji Hospital, Huazhong University of Science & Technology, Wuhan, China; ^3^ Branch of National Clinical Research Center for Metabolic Diseases, Hubei, China

**Keywords:** beiging, brown/beige fat, UCP1, thermogenesis, obesity

## Abstract

Brown and beige adipose tissues possess the remarkable capacity to convert energy into heat, which potentially opens novel therapeutic perspectives targeting the epidemic of metabolic syndromes such as obesity and type 2 diabetes. These thermogenic fats implement mitochondrial oxidative phosphorylation and uncouple respiration to catabolize fatty acids and glucose, which leads to an increase in energy expenditure. In particular, beige adipocytes that arise in white adipose tissue display their thermogenic capacity through various noncanonical mechanisms. This review aims to summarize the general overview of thermogenic fat, especially including the UCP1-independent adaptive thermogenesis and the emerging mechanisms of “beiging”, which may provide more evidence of targeting thermogenic fat to counteract obesity and other metabolic disorders in humans.

## Introduction

Because of the alarming prevalence of sedentary work and high-calorie diets, excessive energy is stored in the form of triglycerides in white adipose tissue (WAT), leading to a high incidence of obesity worldwide. Although the specific pathogenesis of obesity has not been elucidated, the main determining factor is the imbalance of long-term energy metabolism: increased energy intake and/or reduced energy expenditure (1). Suppressing appetite and gastrointestinal absorption by medical and surgical interventions are currently the main interventions to reduce energy intake, but the efficacy is limited. However, prolonged calorie restriction (CR) results in weight loss which promotes metabolic adaptation and decreases energy expenditure leading to an energy deficit (2). Physical training is another choice for energy expenditure. Nevertheless, obese patients generally have poor adherence to regular exercise, instead, the energy expenditure increases their compensatory dietary intake and sedentary time. Thus, it is urgent to find an alternative therapeutic treatment to counteract obesity (3). For the past few decades, brown and beige adipose tissues have been researched due to their distinctive characteristic to dissipate energy into heat. Functional brown adipose tissue (BAT), discovered in adult humans using 18F-flurodeoxyglucose positron emission tomography/computed tomography (18F-FDG PET/CT) (4), specifically targets thermogenic fat and provides a potential strategy to combat obesity and related complications.

## Overview of Brown and Beige Fat

In 2009, several human studies demonstrated that functional BAT is also present in adults (5, 6), which refutes the previous assumption that BAT disappears in the human body at an early age. Histologically, human brown adipocytes were found to contain multilocular smaller lipid droplets and plenty of uncoupling protein 1 (UCP1)-rich mitochondria, which are inextricably linked to its main function of thermogenesis. (**Table 1**) Moreover, there is a high uptake of glucose in the supraclavicular, cervical, paravertebral, and supra-adrenal regions through 18F-FDG PET/CT scan, especially after activation by cold exposure (5, 6).

**Table 1:** Characteristics of different kinds of adipocytes.

	White adipocytes	Brown adipocytes	Beige adipocytes
**Location**	Subcutaneous and visceral	Interscapular (infants)	WAT depots
		supraclavicular, cervical (adults)	(mainly in inguinal)
**Morphology**	Unilocular lipid droplets	Multilocular lipid droplets	
	Few mitochondria	Abundant mitochondria	
	Abundant lipid storage	A few lipid storage	
**Lineage**	Multiple lineage	Myogenic lineage	Multiple lineage
	(PDGFRα+)	(Myf5+)	(PDGFRα+)
**Genetic**	UCP1-	UCP1+	UCP1+ (activated)
**Functions**	Store and release energy	Adaptive thermogenesis	dissipate energy into heat upon stimulation

BAT is formed during the embryonic stage and develops from the myf5+ lineage, consistently with skeletal muscle. BAT is mainly in the interscapular region in human infants and in the supraclavicular and neck regions in adults, with some additional localizations mentioned above (5, 7). A rich capillary bed provides sufficient oxygen and substrate during the regulation of cellular oxidation while dense sympathetic innervation works as an efferent nerve to send signals that focus on innervating and activating brown adipocytes (8, 9). Although active BAT is not abundant in adult humans, it plays a substantial role in energy metabolism depending on numerous thermogenic genes (10, 11). The improvement of PET/CT analysis also shows the metabolic capacity in human BAT is substantially higher than usually reported (12). Of note, although some samples of adult human BAT in neck share similarities with classical rodent BAT (4), more studies tend to the view that human BAT cells have a similar molecular signature with the beige fat cells of rodents (13, 14).

Despite the morphological and functional resemblance to brown adipocytes, beige adipocyte precursors are interspersed within WAT depots, especially in the inguinal region (15). These precursor cells are Myf5- but PDGFRα+ (16, 17). However, the expression of thermogenic genes in beige adipocytes is relatively low at basal, whereas it is induced by cold exposure or specific pharmacological stimulations, such as β-adrenergic stimulants or peroxisome proliferation-activated receptor γ (PPARγ) agonists, to a level equivalent to conventional brown adipocytes with good phenotypic plasticity (7–9, 18). Recently, a type of MyoD+-derived beige adipocyte has been discovered and termed as glycolytic beige adipocyte due to its characteristic of an enhanced glucose oxidation (19). Importantly, this beige fat contributes to cold adaptation in the absence of β3-adrenergic receptor (β3-AR) signaling, which is different from canonical beige fat and regarded as an innovative subtype of thermogenic fat (19–21). Under certain conditions, brown and beige adipocytes generate heat through canonical non-shivering thermogenesis (NST), which is an adaptive response to cold exposure or a high-calorie diet and differs from shivering thermogenesis induced by skeletal muscle (22). Of note, a rare subpopulation of adipocytes has been identified recently both in mice and humans through single-nucleus RNA-sequencing, which regulates BAT thermogenic capacity in a paracrine manner and may be a whole new area of research (23).

BAT quantity and quality differs among individuals. Some studies have indicated that active BAT is more frequently detected in women than men, and gradually decreases with ageing (24). The activity of BAT is inversely correlated to body mass index and glucose levels (25, 26). Thus, enhancement of BAT activation is more imperative to obese patients. Previous rodent studies showed that glucocorticoids have a powerful inhibitory effect on BAT-induced thermogenesis (27), while gonadal hormones (whether androgen or estrogen) may contribute to the differentiation of brown adipocytes (28, 29). A recent study indicated that ESR1, the gene encoding the estrogen receptor, is a requisite for both BAT mitochondrial remodeling *via* mitochondrial fission protein and thermogenesis *via* UCP1, which may reflect the mechanism of sexual dimorphism in BAT abundance and activity (30). Another earlier study showed that female rats were more cold-sensitive and had a high threshold temperature for cold-induced thermogenesis (around 22°C in females compared to 18°C in males), which may also be attributed to the higher BAT activity among women (31). As a cell fate switching, protein PR domain containing 16 (PRDM16) not only plays a decisive role in controlling the differentiation and maintaining the characteristics of brown adipocytes, but also robustly stimulates the formation of beige adipocytes in subcutaneous adipose depots (32, 33). Notably, adipocyte-specific deletion of PRDM16 leads to the absence of functional beige adipocytes, concomitant with hepatic steatosis, as another complication of obesity (34, 35). Furthermore, it has been found that cold adaptation relieves the inhibition of PRDM16 expression by down-regulating miR-133, which is highly expressed in adult skeletal muscle satellite cells and may be an effective target to recruit more active brown adipocytes (36).

The inherent ability of BAT on thermogenesis and accelerating glucose metabolism provides potential therapeutic targets to protect against obesity and its associated complications (37). BAT activation contributes not only to lower blood glucose levels (38), but also to an increase of lipolysis and the level of high density lipoprotein (HDL) cholesterol, which exerts positive effects on lipid metabolism (10, 39). In addition, as an endocrine organ, BAT secretes adipokines to keep metabolic health, including insulin-like growth factor 1 (IGF-1), neuregulin 4 (NRG4), and bone morphogenetic proteins (BMPs) (40). For instance, BAT-derived adipokine 12,13-dihydroxy-9Z-octadecenoic acid (12,13-diHOME) is increased in the intracellular and circulating levels by cold stimulation, which enhances the uptake of free fatty acids (FFAs) into brown adipocytes to facilitate thermogenesis (41). BAT-derived 12,13-diHOME also enhances cardiac function while the levels of 12,13-diHOME are decreased in human patients with heart disease (42), consistently with another cohort study which indicates a beneficial effect of BAT to cardiometabolic health (43). Multiple adipokines play a role in the crosstalk between BAT and other tissues, devoting to signal transmission, physiological regulation, and energy homeostasis (44). However, thermogenic fat also interacts with some pathological conditions. Human and murine obesity will enhance adipose tissue inflammation *via* direct adhesion of inflammatory macrophages, which cause the impaired beige adipogenesis (45). A signaling pathway between thermogenic fat and intestinal epithelial cells regulates intestinal disease tolerance under the condition of adrenergic activation (46). In the state of cancer cachexia, thermogenic fat is activated and whole body energy expenditure is enhanced as a maladaptive response to cachexia-induced anorexia (47, 48). Functional regulation of thermogenic fat may be involved in a promising approach to ameliorate cachexia in cancer patients. All the studies indicate that thermogenic fat plays a crucial role in a variety of pathophysiological states.

## Canonical UCP1-Dependent Thermogenesis

**Figure 1:**
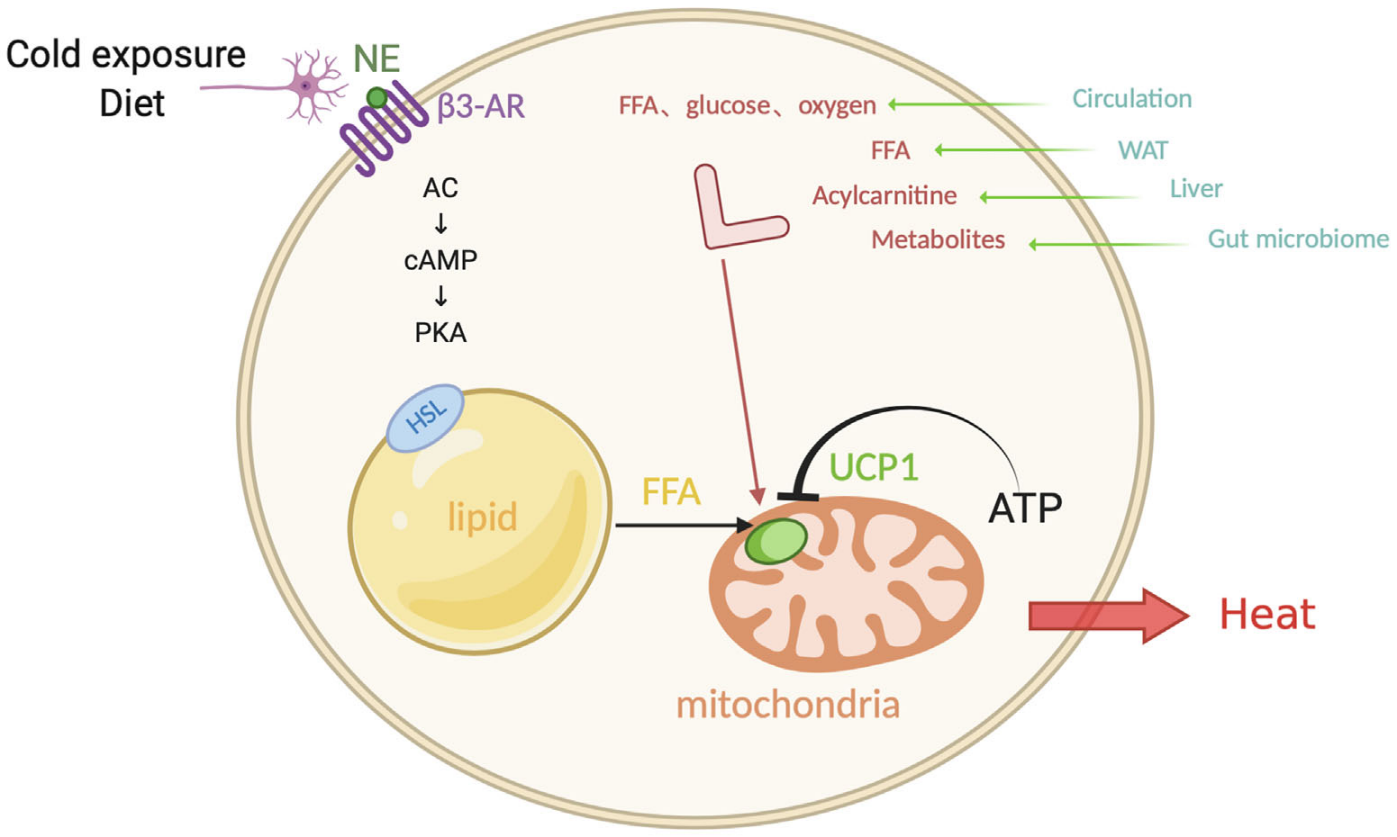
Canonical non-shivering thermogenesis. In response to cold exposure and overeating, norepinephrine (NE) binds to β3-adrenoceptor (β3-AR), initiating adenosine cyclase-cAMP-protein kinase A (AC-cAMP-PKA) pathway. Subsequently the hormone-sensitive lipase (HSL) on the surface of lipid droplets is phosphorylated and the triglycerides translate into free-fatty-acid (FFA) after hydrolysis, which activates uncoupling protein 1 (UCP1) that inhibited by ATP, as a substrate for the following β-oxidation in the mitochondrial matrix. Additionally, interorgan communication provides more available substrates and fuels from exogenous sources, such as glucose, oxygen, FFA, acylcarnitine, metabolites, which come from the circulation, WAT, liver, and gut microbiome. Figure created using BioRender (https://biorender.com/).

For both experimental animals and humans, cold exposure is a powerful trigger for the activation of brown and beige fat. It is interesting to note that cold-exposure-induced epigenetic programming of the sperm grants hyperactive BAT to the offspring (49). In this process, the hypothalamus and brain stem respond to the hypothermic environment and release norepinephrine (NE). NE then binds to β3-ARs on the membrane of brown adipocytes (50, 51)(**Figure 1**). Subsequently, activated adenosine cyclase-cAMP-protein kinase A (AC-cAMP-PKA) signaling pathway induces a cascade reaction, which results in the phosphorylation of certain proteins on the surface of intracellular lipid droplets, such as hormone-sensitive lipase (HSL) and perilipin 1 (PLIN1), followed by the hydrolysis of triglycerides and the release of FFAs. FFAs then activate UCP1, which is located on the mitochondrial inner membrane and primarily expresses in BAT. As an anion/proton symporter, UCP1 transports fatty acids into mitochondria for subsequent β-oxidation. Purine nucleotide ATP has an inhibitory effect on UCP1 while FFAs compete this effect (52, 53). However, the accomplishment of UCP1 activation is dependent on sufficient FFAs, whose level should be at least 100 times higher than ATP, suggesting that the activity of UCP1 is constantly inhibited under normal physiological conditions (53, 54). FFAs, especially long-chain fatty acids, are firmly bound to stimulate UCP1 and thereby mitochondrial respiration, which generates the proton-motive force (△P) to transport protons from mitochondrial intermembrane space into the matrix and provide energy to complete the ADP + Pi-ATP reaction with ATP synthase. Meanwhile, the overall increase in ATP/ADP ratio and △P will conversely in turn restrain mitochondrial respiration, as a negative feedback response to the uncoupling effect (51, 55).

Evidence shows that BAT works as a “metabolic sink” in human bodies. When activated by cold exposure, BAT uptakes FFAs and glucose as fuels. In addition to the above mentioned FFAs from lipid droplets, sympathetic nerve end-released NE induces the release of lipoprotein lipase (LPL) into circulation, which degrades chylomicrons and very low density lipoprotein (VLDL), followed by the generation of FFAs. In addition, glucose is another important substrate which is uptaken by BAT. Cold stress also activates the enzyme 12-lipoxygenase in BAT and then secretes an oxylipin 12-HEPE, which can shuttle glucose into brown adipocytes (56). There is a high demand of oxygen content for combusting FFAs and glucose, which is supplied from adjacent blood that absorbs the released heat. As a result, chronic cold acclimation arouses the overall metabolic capacity of BAT, which is the so-called process of BAT recruitment.

In the process of canonical non-shivering thermogenesis, thermogenic adipocytes are not the only fuel source; it also involves an interorgan communication (22). Cold exposure also promotes lipolysis in WAT, and the FFAs released into the circulation are directly utilized as an energy replenishment for heat production or are shuttled into the liver (57). In the liver, the activation of hepatocyte nuclear factor 4α (HNF4α) by FFAs promotes the expression of mitochondrial enzyme CPT1, which is involved in acylcarnitine synthesis. The synthetic acylcarnitine is another circulating substrate for BAT combustion (58). The loss-of-function experiment of the inability of hepatic acylcarnitine synthesis impairs adaptive thermogenesis as acylcarnitine application in aged mice is sufficient to prevent ageing-related hypothermia. Moreover, alternative bile acid synthesis pathway promotes the production of bile acids and shapes the intestinal microbiome, whose circulating metabolites promote the adaptive thermogenesis and energy expenditure (59). These recent findings imply that peripheral energy sources play a potential role in the canonical non-shivering thermogenesis, which involves a crosstalk between organs.

## UCP1-Independent Adaptive Thermogenesis

Undoubtedly, non-shivering thermogenesis(NST) plays a central role in the whole-body energy homeostasis. It is noticed that deficiency of UCP1, which is primarily thought to be necessary, or inability of adipose fatty acid oxidation in mice did not cause an obvious trend of obesity at thermoneutrality, suggesting that there are alternative thermogenic mechanisms (60).

It has been demonstrated that cold sensitivity in UCP1 knockout mice is completely restored in response to PRDM16 activation (61). The reason is that fat-selective PRDM16 overexpression in UCP1 knockout mice leads to a recruitment of beige adipocytes and produces more systemic energy output compared with the UCP1 knockout littermates. In addition, whether UCP1 is knocked out or not, the glucose tolerance and insulin sensitivity are significantly improved in PRDM16 transgenic mice under the high-fat diet state compared to the control mice. Hence, UCP1-deficient mice also possess a positive metabolic phenotype indicating that beige fat biogenesis driven by adipose-specific overexpression of PRDM16 protects from cold-induced hypothermia, diet-induced obesity, and glucose intolerance (33, 61).

Recently, UCP1-independent mechanisms involved in NST have been shown to include sarco/endoplasmic reticulum Ca2+-ATPase2b (SERCA2b)-mediated Ca2+ cycling and creatine-driven substrate cycling, however, they both mainly exist in beige fat due to a high demand of ATP (61–63). In response to cold stimuli, NE activates α1-adrenergic receptor (α1-AR) and β3-AR, which triggers intracellular Ca2+ flux. SERCA2b regulates Ca2+ uptake into the endoplasmic reticulum while ryanodine receptor 2 (RYR2) and inositol trisphosphate receptor (IP3R) promote its release, a process of futile cycling involved in ATP hydrolysis and thermogenesis. Meanwhile, SERCA2b-RYR2 pathway increases mitochondrial Ca2+ by calcium uniporter, which activates pyruvate dehydrogenase (PDH) and ATP synthase. Furthermore, beige adipocytes expend glucose as the primary fuel source through enhanced glycolysis for ATP generation in the context of UCP1 ablation and work as a “glucose-sink” to positively regulate glucose tolerance independent of body-weight loss. Thus, the loss of UCP1 in beige fat is able to be compensated, and this compensation is found not only in mice and humans, but even in pigs, a species that naturally lacks functional UCP1 (64).

Consistently, creatine-driven substrate cycling, involving its phosphorylation by mitochondria-localized creatine kinase (Mi-CK) and de-phosphorylation by tissue-nonspecifc alkaline phosphatase (TNAP), also plays a pivotal role in energy homeostasis in the lack of UCP1 (62, 65). Genes of creatine metabolism are induced after cold exposure through stimulation of creatine kinase activity, which promotes mitochondria respiration. Drug intervention to reduce creatine levels in beige fat attenuates lipid metabolism and systemic energy output, while core body temperature of UCP1−/− mice is difficult to maintain. These phenomena prove that creatine metabolism contributes to beige adipose-specific energy expenditure and thermogenesis. It should be noted that once long-term β3-adrenergic stimulation initiates “beiging”, creatine-driven substrate cycling would co-exist with canonical thermogenesis to orchestrate the effects of basal metabolic rates (66). What’s more, owing to the similar molecular characteristics between human brown adipocytes and murine beige adipocytes mentioned above, it has been identified that creatine regulates the respiration of isolated human brown adipocytes, which could open up possibilities to manipulate thermogenesis in patients with obesity and other metabolic diseases. In conclusion, these non-canonical thermogenic mechanisms may provide further therapeutic possibilities, especially for elderly and obese patients who may lack UCP1 in adipose tissue.

## Beiging

BAT activation negatively correlates with body weight (18). An early study reported that the metabolic response to NE infusion and the adaptive thermogenesis are attenuated in obese patients with family history (67). It suggests that obese patients find it more difficult to lose weight through BAT thermogenesis. In recent years, it has been documented that thermogenic fat can be induced in WAT in both mice and humans. It opens up an intriguing treatment intervention, especially for obese patients with a large surplus of WAT (68). In addition to physiological stimuli such as chronic cold exposure, pharmacological treatment including PPARγ agonist and β-adrenergic stimulation contribute to “beiging”. Synthetic PPARγ ligands thiazolidinedione drugs (TZDs) has been shown to induce “beiging” by prolonging the half-life of PRDM16 (69). One of the natural molecules, flavonoids, induce “beiging” by activating AMP-activated protein kinase (AMPK)-PGC1α/Sirtuin 1 (SIRT1) and PPARγ signaling pathways. Furthermore, they also promote the differentiation of brown preadipocytes and inhibit their apoptosis (70, 71). In addition, there are a subset of common natural compounds which promote “beiging” in WAT (**Table 2**). For instance, capsaicin and dietary fish oil induce “beiging” in WAT *via* activation of transient receptor potential vanilloid 1 (TRPV1) (75, 76), which arouse interests about the effect to attenuate fat accumulation for someone who prefers to take chili and fish oil. As a popular beverage, green and black tea contains catechins and theaflavins that promote beige adipogenesis by activating AMPK (78, 79). Other regulatory factors which are involved in beige fat development include fibroblast growth factor 21 (FGF21), bone morphogenetic protein 7 (BMP7), irisin, and cyclooxygenase 2 (COX-2) (84, 85). Specifically, FGF21 plays a physiologic role in recruiting beige fat cells in WAT depots by increasing transcription factor PGC1α level (86). Moreover, muscle-derived irisin, which is regulated by PGC1α, stimulates beige adipogenesis and exercise induces the release of irisin into the blood to drive the process of “beiging” (87).

**Table 2:** Summary of dietary compounds involved in beige adipogenesis.

Molecule name	Main source	Target		Action mechanism		Reference
Curcumin	Turmeric		β3-AR	↑ β3-AR and plasma NE		(72)
Resveratrol	Grape skins		AMPKα1	AMPKα1-SIRT1 activation		(73)
Berberine	Coptis chinensis		AMPK	AMPKα1-PGC1α activation		(74)
				↑ TH and sympathetic outflow		
Capsaicin	Red chili pepper		TRPV1	CaMKII-AMPK-SIRT1 activation		(75)
EPA and DHA	Fish oil		TRPV1	Sympathetic nerve activation		(76)
				Anti-inflammatory action		
Menthol	Menta		TRPM8	PKA phosphorylation-mediated		(77)
				UCP1 activation		
Catechins and	Green tea and		AMPK,PPARα	AMPK activation		(78, 79)
theaflavins	black tea					
Ginsenoside	Panax ginseng		AMPK	AMPK activation		(80, 81)
Honokiol	Magnolia		ERK	ERK activation		(82)
Naringenin	Citrus		AMPK,PPARα and γ	↑ AGTL,PGC1α and β		(83)

AMPK, AMP-activated protein kinase; ATGL, adipose triglyceride lipase; β3-AR, β3-adrenoceptor; CaMKII, Ca2+/calmodulindependent protein kinase II; ERK, extracellular signal-regulated kinase; NE, norepinephrine; PGC-1 α/β, peroxisome proliferator-activated receptor gamma coactivator l alpha/beta; PKA, protein kinase A; PPAR, Peroxisome proliferator-activated receptor; SIRT1, sirtuin 1; TH, tyrosine hydroxylase; TRPM8, transient receptor potential melastatin 8; TRPV1, transient receptor potential vanilloid 1; UCP1, uncoupling protein 1. The arrows mean the elevating level of related substance and process.

**Figure 2:**
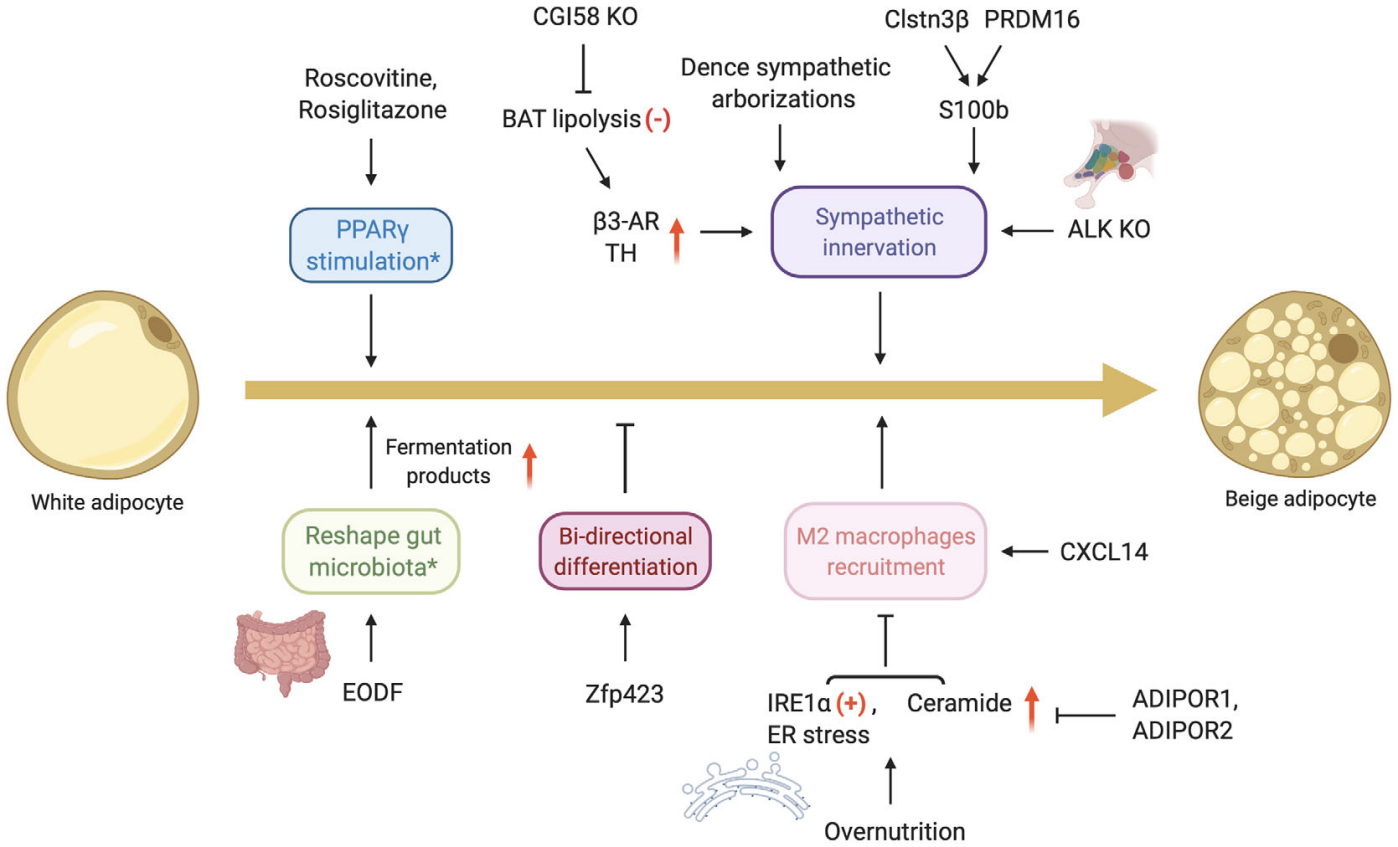
Emerging mechanisms involving in “beiging” of WAT. ADIPOR, adiponectin receptors; ALK, anaplastic lymphoma kinase; AR, adrenergic receptor; CGI-58, comparative gene identification-58; Clstn3β, calsyntenin 3β; CXCL14, C-X-C motif chemokine ligand-14; EODF, every-other-day fasting; ER, endoplasmic reticulum; IRE1α, inositol-requiring enzyme 1α; PPARγ, peroxisome proliferation-activated receptor gamma; PRDM16, protein PR domain containing 16; TH, tyrosine hydroxylase; Zfp423, C2H2 zinc-finger protein. The content in the boxes corresponds to the relevant mechanism and the asterisk indicates the progress of “beiging” does not involve β-adrenergic pathway. The signs (**+/-**) in red represent the activation/inhibition status while the arrows in red mean the elevating level of related substance. Figure created using BioRender (https://biorender.com/).

Along with several special molecules and regulators, physical exercise also contributes to “beiging”. In mice, exercise training promotes the mitochondrial synthesis, activity, and glucose uptake in subcutaneous white adipocytes, which are dependent on endothelial nitric oxide synthase (eNOS) (88). Furthermore, transplanting subcutaneous WAT of the exercised mice into the sedentary mice obviously improved the glucose uptake of skeletal muscle and the overall metabolic balance (89). Importantly, the circulating adipokine concentration and their expression in adipose tissue is altered in both human and mouse after exercises (89). These adipokines may play a prominent role in the dramatic metabolic effects which need to be further identified. In humans, the origin of beige fat is still controversial and whether “beiging” increases energy expenditure remains unclear. The capacity of “beiging” in humans specifically needs further investigation (68). Of note, improved flow cytometry is available to sort and quantify UCP1+ beige adipocytes after various interventions and as an effective method (90). Here, we focus on a few novel perspectives on the mechanisms of “beiging”, which have been discovered so far (**Figure 2**).

### A Distinct UCP1+ Adipocyte Induced by Roscovitine

Roscovitine is an inhibitor of cyclin-dependent kinase (CDK) and prevents S273 phosphorylation of PPARγ, which in turn induces beige adipocyte development, increases energy expenditure, and mitigates insulin resistance (91). These adipocytes express UCP1 and are distinct from canonical beige adipocytes both in morphology and mRNA signature. For instance, Cdsn, Rhbg, and Gpx8 are enriched in roscovitine-induced adipocytes, which are paucilocular and different from multilocular beige adipocyte. Besides, they are similar to the beige adipocytes induced by rosiglitazone. This is probably because these two drugs share the same mechanism of PPARγ stimulation rather than the β-adrenergic pathway. These phenomena indicate that there are various subtypes of thermogenic adipocytes. They have been found to include glycolytic beige adipocyte and UCP1+ adipocytes upon transforming from white adipocytes or differentiation from multiple developmental origins (19, 20). Future studies will possibly focus on the mechanism of the development of different UCP1+ adipocytes, which may contribute to the adaptive thermogenesis.

### Every-Other-Day Fasting (EODF)-Induced Gut Microbiota Remodeling

EODF, as a form of intermittent fasting, has been reported to initiate “beiging” in the subcutaneous inguinal WAT by reshaping gut microbiota and contributing to metabolic homeostasis (92). Long-term EODF leads to a gradual change in the gut microbiota composition, which results in an augment of fermentation products such as acetate and lactate. They act as “beiging” agonists to dramatically upregulate the expression of UCP1 and stimulate “beiging” in WAT independent on β3-AR and FGF21 signaling pathway. Intriguingly, EODF diet does not influence cumulative food intake, which reveals the effect of weight loss is the increased energy expenditure. Conceivably, for people with poor compliance of EODF regimen, further exploration on the mechanisms of EODF-induced “beiging” is particularly conducive to capturing new therapeutic methods to combat obesity and metabolic diseases.

However, the effect of microbiota depletion on “beiging” is controversial. Some studies show microbiota depletion stimulates the development of beige fat and gains metabolic improvements whereas another study presents negative effects that microbiota depletion reduces the process of “beiging” (93, 94). Although the results are different, the fact is that microbiota-fat signaling pathway may be correlated to beige fat recruitment. Nevertheless, gut microbiota exist in the body, so that the applications of microbiota depletion in humans would be limited in clinical studies.

### Determination of White or Beige Adipocyte by Zfp423

As a transcriptional regulator of white adipogenesis, C2H2 zinc-finger protein Zfp423 plays a critical role in maintaining the phenotype of WAT (95). A 2016 paper from the Pfeifer Lab indicates that in the progress of BAT whitening, a reversible process characterized by mitochondrial dysfunction, lipid droplet accumulation, and decreased vascularity (96), Zfp423 expression increases and effectively inhibits the expression of some thermogenic genes including Ucp1 (97). There also shows a negative correlation between Zfp423 and the activity of Ebf2 and PRDM16, which drive the activation of thermogenic program and keep the identity of brown and/or beige fat (98). In mice lacking Zfp423, β-adrenergic stimulation strengthens the effect of Zfp423 deletion to transform white adipocytes to beige adipocytes in inguinal and gonadal fat, leading to the reverse of diet-induced obesity and glucose metabolism disorders. From an evolutionary viewpoint, intervention on Zfp423 highlights the potential of bi-directional interconversion between white and beige adipocytes. It should be noted that chronic cold exposure induces beige adipocyte formation while subsequent heat adaptation converts them into the same cells as white adipocytes in morphology and gene phenotype. Moreover, these specific adipocytes can transform into beige adipocytes once again after further cold stimulus, which indicates a promising adaptive plasticity of white adipocytes (99, 100). In sum, future efforts focused on certain determination factors which would regulate the bi-directional differentiation of white-beige adipocytes should be made to find new potential targets for ameliorating obesity and sustaining metabolic balance.

### M2 Macrophages Polarization and Recruitment

An adipokine, chemokine C-X-C motif chemokine ligand-14 (CXCL14), has been identified to be released by activated brown adipocytes, which mediates a brown fat-macrophage communication and drives the adaptive thermogenesis by promoting both BAT activation and “beiging” in WAT (101). This positive feedback effect depends on CXCL14 induced activation of type 2 cytokine signaling and recruitment of macrophages, especially M2 phenotype, in WAT. However, the specific mechanisms by which M2 macrophages’ recruitment improves the adaptive thermogenesis remain controversial. One of the underlying causes was thought to be the secretion of catecholamines triggered by the enhanced activation of adipose tissue macrophages (ATMs) (102), although this has since been refuted (103).

Additionally, the most conserved endoplasmic reticulum (ER) stress sensor inositol-requiring enzyme 1α (IRE1α) governs M1-M2 macrophage polarization and drives obesity by influencing BAT activity and “beiging” in WAT (104). Some obesogenic factors, such as overnutrition, activates IRE1α in ATMs and increases ER stress, which disequilibrates the M1-M2 polarization and decreases M2 recruitment. Nevertheless, IRE1α abrogation reverses the imbalance and promotes M2 activation of ATMs, leading to “beiging” in WAT and augmentation of energy output. Overnutrition and obesity also increase the content of adipose sphingolipids, whose intermediates include ceramide and its metabolites (105). Whole-body and fat-specific inhibition of ceramide synthesis induces “beiging” and M2 macrophage recruitment preferentially in subcutaneous WAT, consistently with an improvement in thermogenic gene expression, mitochondrial function, and energy expenditure. Physiologically, two subtypes of adiponectin receptors, ADIPOR1 and ADIPOR2, possess intrinsic activity of ceramidase and can hydrolyze ceramide (106), which may cause similar metabolic effects of ceramide synthesis inhibition. Hence, it reveals that M2 macrophage polarization and recruitment orchestrating adaptive thermogenesis have potential therapeutic benefits on obesity.

### Sympathetic Innervation on Adipose Tissue

A recent study has indicated that there is still general thermogenic capacity without cold stimulation in mice when BAT lipolysis is inhibited by tissue-specific knockout of comparative gene identification-58 (CGI-58), which is a vital lipolysis catalyst (107). The mechanism to compensate for this defect includes a development of beige fat, which depends strongly on the sympathetic innervation through elevating the level of tyrosine hydroxylase (TH), promoting the production of dopamine and β3-ARs in WAT. Because of the recruited BAT and beige fat, most of the circulating nutrients, such as glucose, flow into these thermogenic tissues for heat production while fewer nutrients enter other tissues for pathological accumulation. Moreover, the CGI-58 knockout mice in the fed generally show a higher body temperature and energy output during cold exposure than the littermates. Thus, selective inhibition of BAT lipolysis by CGI-58 ablation contributes to beige fat development through an improved sympathetic innervation in WAT, which results in a steady-state metabolism.

Dense sympathetic arborizations in WAT have been directly observed under microscope by the volume fluorescence-imaging technique. They are embedded in WAT and closely positioned to most adipocytes, which are notably indispensable for “beiging” induced by cold acclimation (108). In mice with an ablation of sympathetic arborization, it failed to increase the expression of thermogenic genes and induce the formation of multilocular adipocytes in inguinal WAT (iWAT). Furthermore, catecholamines released from sympathetic arborization and β3-ARs play a critical role on the progress of “beiging” during cold exposure. Of note, a recent discovery from Spiegelman’s group has identified calsyntenin 3β (Clstn3β), which is a thermogenic fat-specific protein located in the endoplasmic reticulum. It enhances the secretion of S100b in response to cold, which promotes sympathetic innervation and thereby improves non-shivering thermogenesis (109).

Furthermore, another factor, anaplastic lymphoma kinase (ALK), has been identified to be a thinness gene candidate (110), which is derived from hypothalamic paraventricular nucleus. Its deletion results in an increase in NE level and enhancement in lipolysis both in BAT and WAT (involvement of sympathetic innervation). Consistently with the effect of NE-induced “beiging” (111), the thermogenic and mitochondrial gene expression is also substantially upregulated in WAT. Overall, ALK is a negative regulator of “beiging” and lipolysis *via* sympathetic tone. Its inhibition could be a promising strategy for maintaining energy homeostasis and therefore resisting weight gain.

## Potential Treatment Trends and Prospects

Classical non-shivering thermogenesis, which is UCP1-dependent, contributes to energy dissipation by boosting mitochondria-mediated disposal of glucose and lipids, which benefits obesity and its related complications such as diabetes and hyperlipidemia. As a main fuel source for heat generation, lipids also exert regulatory effects on mitochondrial dynamics and bioenergetics, thermogenic gene expression, thermogenic adipocytes differentiation, signal transduction, and interorgan communication (22), thereby, a comprehensive understanding of the diversity of lipid functions in thermogenic fat is necessary to be further explored for the treatment of human obesity.

The most direct way to elevate thermogenic effect is to potentiate the quality and quantity of active thermogenic fat. As previously known, cold exposure has an intense activating effect on brown and beige fat. In a human study, daily cold exposure (10°C; 2h/d) for four consecutive weeks promotes BAT oxidative capacity and attenuates shivering intensity and thermogenesis in skeletal muscles, which demonstrates a reciprocal effect between BAT and skeletal muscle and an enhanced contribution of BAT activation to cold-induced heat production (112). However, chronic cold adaptation challenges the clinical feasibility to engage in obese patients because of the general preference for thermal comfort. Selective β3-AR agonists also activate thermogenic fat and promote “beiging”, yet are concomitant with poor bioavailability, not only because of less expression and lower efficacy of β3-AR agonists in human white adipocytes, but also due to some serious side effects, especially on the cardiovascular system (113). In point of fact, human brown adipocytes primarily express β2-AR, which co-localize with UCP1 and present a novel pharmacological target to stimulate human BAT (114). A similar situation is for the PPARγ agonists, as pioglitazone also causes various side effects such as exacerbations on congestive heart failure, bladder cancer, and bone fractures (115). One of the artificial FGF21 analogs, LY2405319 (LY), has been trialed in patients with obesity and type 2 diabetes, and results in significant improvements in dyslipidemia, body weight, and fasting insulin (116, 117). However, bone loss is a safety concern of FGF21 analog, which has been reported in rodents, and its potential effect on atherogenesis in humans may be worth considering (118).

Thermogenic fat recruitment could be another approach of therapeutic interventions to treat obesity. Preadipocytes “beiging” in WAT is a pivotal recruitment source of thermogenic fat. As mentioned above, some pharmaceuticals and common natural compounds promote the progress of “beiging”, and several regulators influence “beiging” in WAT involving the mechanisms of sympathetic innervation, M2 macrophages recruitment, gut microbiota remodeling, etc. All these show “beiging” in WAT is a holistic process with interorgan communication and provides an extensive research field of bi-directional differentiation of white and thermogenic adipocytes. Moreover, initial mesodermal induction of human pluripotent stem cells (hiPSCs) activate the differentiation of adipocytes with a thermogenic phenotype through a distinct protocol (18). It is valuable to provide an unlimited cell source focusing on human thermogenic adipocyte recruitment, which is still under investigation (18, 119). Bone marrow-derived cells (BMCs) could infiltrate into iWAT through circulation and differentiate to white adipocytes under high fat diet (HFD) feeding or UCP1+ thermogenic adipocytes following β3-AR activation (120). Hence, BMCs act as nonresident progenitors contributing to the adipose tissue thermogenesis. In addition to hiPSCs and BMCs, adipocyte precursors have drawn attention to develop intracellular gene-editing methods for drug screening and transplantable therapeutics (121). As is expected, BAT transplantation for 2-3 months strikingly ameliorates HFD-induced insulin resistance and hyperlipidemia, lowers body weight, and improves the whole-body energy balance following “dose effect” in mice (122). Moreover, BAT transplantation enhances the gene expression, which is related to FFA oxidation in endogenous BAT, and may potentiate the inherent adaptive thermogenesis (123). Remarkably, implantation of human beige adipocytes into recipient mice generates a similar beneficial metabolic response, enlightening that it may become a feasible intervention for diabetes and obesity treatment (124). CRISPR-engineered technology has been used on human white preadipocytes to create brown-like cells, which are endogenously activated of UCP1. Transplantation of these cells improves metabolism in diet-induced obese mice and also activates endogenous BAT (125). Nevertheless, there are fewer thermogenic genes in the transplanted BAT. Likely owing to the absence of propriate sympathetic stimulation, and the ubiquitous transplantation rejection, BAT-related transplantation needs to be focused on in further research (123). Taken together, although drugs that activate thermogenic fat have no widespread application due to their side effects and insufficient feasibility, the induced differentiation of pluripotent stem cells and adipose precursors from various cell linages into thermogenic adipocytes and the transplantation of thermogenic fat as emerging research field could hold promise as a new class of therapeutics.

Another point worth noting is that there may be multiple subtypes of thermogenic adipocytes, which originate from different precursor cells, are regulated by distinctive molecular switches, and have unique biological roles besides thermogenesis. UCP1 is not indispensable because cold sensitivity can be compensated by PRDM16 activation and recruitment of beige adipocytes with UCP1-independent thermogenic mechanisms. While PRDM16 is not always the molecular switch of thermogenic adipocytes, Gabpα also drives myogenic beige fat differentiation which burns mainly sugar (19). These show the thermogenic adipose cell heterogeneity of developmental origin and regulation of cellular metabolism with canonical or noncanonical mechanisms.

## Conclusion

In sum, thermogenic fat draws considerable attention owing to the contribution to systemic energy homeostasis through dissipating energy into heat. More studies have shown various subtypes of thermogenic adipocytes, different mechanisms of UCP1-independent thermogenesis, and “beiging” with interorgan communication, which enrich the knowledge of thermogenic fat and raise the possibility for clinical approaches for the management of obesity and related complications.

## Author Contributions

ZW and YC wrote the manuscript. XY and YC edited the manuscript. All authors contributed to the article and approved the submitted version.

## Funding

This study was supported by the National Natural Science Foundation of China (82070859 to YC, ZW) and a grant from Tongji Hospital in Huazhong University of Science and Technology (Grant No. 2201103295 to YC).

## Conflict of Interest

The authors declare that the research was conducted in the absence of any commercial or financial relationships that could be construed as a potential conflict of interest.
